# The Alliance Negotiation Scale – Therapist Version: Psychometric Properties in a Sample of Portuguese Psychologists

**DOI:** 10.32872/cpe.11477

**Published:** 2024-06-28

**Authors:** Ana Catarina Nunes da Silva, Marta Matos, Helena Carvalho

**Affiliations:** 1 Faculdade de Psicologia, Universidade de Lisboa, CICPSI, Lisbon, Portugal; 2 CIS-IUL – Centro de Investigação e Intervenção Social, ISCTE – Instituto Universitário de Lisboa, Lisbon, Portugal; 3 CIES-IUL – Centro de Investigação e Estudos de Sociologia, ISCTE – Instituto Universitário de Lisboa, Lisbon, Portugal; Philipps-University of Marburg, Marburg, Germany

**Keywords:** negotiation, Alliance Negotiation Scale, Portuguese version, therapist version, psychometric properties, scale validation

## Abstract

**Background:**

The transtheoretical conceptualization of the working alliance and the resultant evaluation tools often overestimate the collaboration between therapist and client, while neglecting the negotiation process. The degree to which therapists and clients can negotiate disagreements regarding goals and tasks is an important indicator in establishing and maintaining the alliance. Even though the negotiation concept is not new, there is still a lack of reliable and parsimonious self-report measures of the construct. The purpose of this study was to translate, execute the cultural adaptation and, also, to perform a preliminary psychometric analysis of the Portuguese form of the therapist version of the Alliance Negotiation Scale (ANS-T_Pt).

**Method:**

Data were collected online from 100 Portuguese psychologists. Two random sub-samples were used to conduct both exploratory factorial analysis and confirmatory factorial analysis. Convergent validity was assessed through comparison with the Portuguese version of the Working Alliance Inventory.

**Results:**

The ANS-T_Pt showed a one-factorial structure, consistent with previous versions, and demonstrated adequate internal consistency. Evidence supporting criterion-related validity was found based on the correlations between ANS-T_Pt and WAI-T scores. The results showed moderate to large associations between the instruments. These results support the usefulness of the scale, construct’s relevance and its transtheoretical nature.

**Conclusion:**

These results are a step forward for Portuguese therapists’ and researchers’ ability to evaluate the bond between client and therapist and to compare results from different countries.

## Highlights

- The Portuguese form of the ANS – Therapist Version showed good psychometric characteristics.
- Negotiation in challenging relationships was positively associated with the therapist experience.
- The measure can be useful not only for research but also for clinical practice and supervision.

The quality of the therapeutic alliance between psychotherapists and clients has been showed, for decades, to be an essential ingredient in promoting therapeutic change, especially as perceived by the client (Flückiger et al., 2012; Horvath et al., 2011). The most used concept of alliance in psychotherapy is based on the working alliance definition by Bordin (1979) who defined it as a collaborative stance between the client and the therapist. The concept of *working alliance* is composed of three aspects: (a) agreement on the therapeutic goals to be reached; (b) agreement on the tasks to be developed; and (c) the quality of the relational bond, which encompasses the affective quality of the relationship between the client and the therapist. Bordin (1979) also hypothesized that different theoretical frameworks would emphasize different aspects of the working alliance, since different theoretical orientation emphasize different tasks and goals.

Indeed, the working alliance has systematically proved to be a solid predictor of the therapeutic results, regardless of the therapists’ theoretical orientation (Safran & Muran, 2000; Zuroff & Blatt, 2006). However, clinicians commonly observe that, especially with clients facing severe psychological conditions like personality disorders, an initially poor working alliance can be repaired and transformed into a positive one (e.g., Safran et al., 2011). This underscores that a robust alliance alone does not guarantee therapy effectiveness; rather, it can result from effective interventions. Working together in therapy eventually allows a good alliance between both parties to develop, especially when able to work on rupture and repair (Råbu et al., 2011; Safran et al., 2011). For some clients, the goal of therapy might even be to develop the ability to be in a close and secure relationship (e.g., Norcross & Wampold, 2018).

Safran and Muran (2006) sustained that the quality of the therapeutic relationship is related to the processes involved in the resolution of conflicts in case they arise, and not determined by the absence of conflicts or lack of collaboration. Therefore, it is important to re-conceptualize the working alliance as a process of a continuous negotiation of the needs of two independent subjects involved in the relationship. The concept should include how far disagreements and tension are processed by and within the therapeutic relationship (Muran et al., 2009; Safran & Muran, 2000). From this perspective, the negotiation process allows for change to occur and it is a central component of the process of change (Doran et al., 2012). Furthermore, considering the association between the process of rupture-repair and the therapeutic results, it is important to understand the underlying and facilitating mechanisms in this process, which leads us to the concept of the *alliance negotiation*.

Alliance negotiation consists of the client and therapist’s ability to solve relational problems and disagreements, in their therapeutic goals and tasks, during therapy (Doran et al., 2016; Safran & Muran, 2006). As a dyadic concept, it holds significant clinical implications across various theoretical frameworks. It is important to emphasize that the dimensions of collaboration and negotiation are not mutually exclusive and offer complementary points of view of the working alliance (Doran et al., 2016). An important body of literature have suggested that alliance negotiation is one of the most important elements of therapy and a common factor for different theoretical approaches (Baier et al., 2020; e.g., Wampold & Imel, 2015; Zilcha-Mano & Ben David-Sela, 2022).

Currently, the measurement of alliance negotiation between clients and therapists is limited to a single self-report instrument, encompassing one scale from the clients' perspective (Doran et al., 2012, 2016) and another from the therapists’ perspective (Doran et al., 2018; Gómez-Penedo et al., 2019). The Alliance Negotiation Scale (ANS) was modelled in structure and form after the Working Alliance Inventory (WAI, Horvath & Greenberg, 1989) which is one of the most used measures of the working alliance.

The ANS was developed to introduce a specific focus on negative aspects of the therapeutic process, particularly addressing the presence and resolution of ruptures in therapy (Doran et al., 2016). Indeed, dealing with different types of difficulties related to the working alliance is essential to the course of therapy (for a review see Doran et al., 2016). The Alliance Negotiation Scale – Therapist Version (ANS‐T) was jointly developed in its North American and Argentinian versions. Considering the transtheoretical and cross‐culturally importance of the concept, a collaborative cross‐cultural effort to create a therapist version was made. The ANS‐T is not an identical translation of the client version. The client version contains 12 items and two factors, while the ANS‐T is unidimensional and contains only nine items. The authors argue that although it would have seemed preferable to have a version of the ANS‐T that more closely mirrored the client ANS (12 items and/or two factors), it was deemed more important to create the most psychometrically sound scale possible (for a detailed description of the scale development see Doran et al., 2018).

Results from both samples support the composition of the ANS‐T and provide initial support for the reliability and validity of the measure (Doran et al., 2018; Gómez-Penedo et al., 2019). Through a principal components analysis procedure, it presented nine unidimensional items and was moderately correlated with therapist‐reported working alliance (*r* = .468, *r* = .51), North American and Argentinian results respectively (Doran et al., 2018; Gómez-Penedo et al., 2019).

Given the dyadic nature of alliance negotiation, having both a client version and a therapist version is essential. In the European-Portuguese context, there already exists a client version of the ANS (Galvão et al., 2019). Therefore, the primary goal of the present study is to introduce and make available the therapist version of the scale.

Considering the importance of the alliance negotiation and its implications for the outcomes of the therapeutic process, the present study seeks to address the absence of a Portuguese form of the therapist version of the ANS. Accordingly, this study aims to translate, perform the cultural adaptation and a preliminary psychometric analysis of the Portuguese form of the therapist version of the Alliance Negotiation Scale (ANS-T_Pt) in a Portuguese sample of therapists. Furthermore, by previously adapting the clients' form of the ANS (Galvão et al., 2019), we enable research into dyadic perceptions of alliance negotiation. This approach facilitates a comprehensive examination of alliance negotiation from both therapist and client perspectives.

## Method

### Participants

One hundred therapists participated in this study. Participants were mostly females (*N* = 85, 85.0%). Mean age was 38.58 (*SD* = 9.82) and ranged between 23 and 63. Sixty six percent of participants had a master’s degree, 24.0% graduated from university, and 10.0% had a PhD. The average of years of clinical experience of the participants of the sample were 12.06 years old (*SD* = 8.68 years old). The therapist with the least experience had one year of clinical practice, while the most experienced referred 35 years of clinical practice. Seventy six percent worked in private practice, 11.0% in a social solidarity private institution, 8.0% in hospitals and 6.0% in primary care facilities (non-excluded response categories). Thirty three percent reported a CBT-based integrative approach, 14.0% a CBT approach, 5.0% psychodynamic approach, 1.0% systemic and 47.0% did not specify their theoretical approach.

Each participant provided with data on two cases – (1) perceived as a good therapeutic relationship (GTR) and (2) perceived as a more challenging therapeutic relationship (CTR). For the clients assigned to GTR, therapists reported that more than half of their clients were female (62.0%). With a mean age of 34.93 (*SD* = 13.00 years old) ranging from 18 to 77 years old. With a medium number of 29 sessions ranging from 2 to 107. Client diagnoses included relational problems (55.0%), depressive disorders (39.0%), anxiety disorders (49.0%), or other clinical syndrome such as an eating disorder or adjustment disorders (10.0%). A subset of the sample was diagnosed with a personality disorder (10.0%), mostly defined as Dependent, Avoidant or Borderline Personality Disorder.

For the clients assigned to CTR, therapists reported that more than half of their clients were female (58.0%). With a mean age of 37.91 (*SD* = 13.02 years old) and range from 18 to 80. With a medium number of 25 sessions ranging from 1 to 160. Client diagnoses were very similar to the ones reported in the GTR group, with mainly anxiety disorders (58.0%), relational problems (56.0%), depressive disorders (41.0%), or other clinical syndrome such as an eating disorder, or adjustment disorders (9.0%). A subset of the sample was diagnosed with a personality disorder (25.0%), mostly defined as Borderline, Narcissistic, Avoidant, Histrionic, or Dependent Personality Disorder.

### Instruments

#### Sociodemographic Data

For the purposes of this research a short questionnaire was created to list the demographic data of the participants. Clinicians indicated their gender, age, nationality, level of education, and also provided information about their theoretical orientation, number of sessions, and their client's age, gender, presenting problems and diagnoses.

#### Alliance Negotiation Scale – Therapist Version

The purpose of the Alliance Negotiation Scale – Therapist Version (ANS-T; Doran et al., 2018; Gómez-Penedo et al., 2019) is to assess the degree of negotiation in the therapeutic alliance, from the therapist’s perspective. It includes nine items. Items are rated on a 7-point Likert-type scale ranging from 1 (Never) to 7 (Always). Therapists are asked to indicate the number that best applies to the way they feel about their relationship with their client. The total average result reflects the therapists’ perception of the degree of negotiation in the therapeutic alliance. The scale computation was done by summing the nine items, with high results indicating a higher level of negotiation. In the present sample, for the purpose of testing reliability and validity two different alliance negotiation variables were computed, according to the two types of cases, both showing excellent and very good internal consistency (α_GTR_ = .89; α_CTR_ = .80) (Kline, 2011).

#### Working Alliance Inventory – Short Form

Concerning the therapeutic alliance, the Working Alliance Inventory – Short Form (WAI-S, Horvath & Greenberg, 1989; Tracey & Kokotovic, 1989), Portuguese version (Machado & Horvath, 1999), was used. The WAI-S is an inventory that assesses the working alliance and is composed by three dimensions regarding the conceptualization of Bordin (1979): bond, agreement between therapist and client on goals and agreement between therapist and client on tasks. Participants reported the frequency of feeling and thoughts in relation to the other element of the therapeutic dyad, on a Likert scale (from 1 “never” to 7 “always”). The short version has 12 items, four for each dimension (Tracey & Kokotovic, 1989). The scale computation was done by summing the items for each sub-scale and for the global scale. Higher results indicate a higher level of strength and quality of the working alliance, from the therapist’s perspective. The internal consistency of this instrument, in this study, for both cases – good therapeutic relationship (GTR) and challenging therapeutic relationship (CTR) – for each sub-scale and the total scale ranged from fair to excellent: Global scale (α_GTR_ = .89; α_CTR_ = .88) which is consistent with the original Portuguese version (α = .89; Machado & Horvath, 1999), Goals (α_GTR_ = .74; α_CTR_ = .77), Tasks (α_GTR_ = .58; α_CTR_ = .60) and Bond (α_GTR_ = .89; α_CTR_ = .81).

### Procedures

Firstly, regarding the translation and cultural adaptation several steps were taken to, following Beaton et al. (2000) guidelines to cross-cultural adaptation of self-report measures. Permission was sought and obtained from Jennifer Doran for the Portuguese adaptation of the measure. The ANS-T (Doran et al., 2018) was, then, translated into Portuguese, by three therapists fluent in Portuguese and English which resulted in three versions. The different versions were compared, and a discussion was held to reach an agreement between the experts. Subsequently, a Portuguese form was back translated into English (retroversion) by an experienced Portuguese psychotherapist highly proficient in English language. The original items were compared with the new items in English, the result of the backward translation (Hambleton et al., 2005), and there were no substantial differences between both versions. Finally, Jennifer Doran approved the back translation. To ensure the clarity of the translated items, a pre-test was conducted with 10 therapists. This process confirmed the clarity of the items (final version can be accessed in the Supplementary Materials).

Secondly, concerning the psychometric study, participants were recruited following two inclusion criteria: a) being a psychologist registered in the Border of Portuguese psychologists and b) having Portuguese nationality. Data was collected on-line using Google forms, and participants were asked for written consent and assured of confidentiality on the first page of the online form and after were presented with the instruments. Data collection was done only once per participant, but there was an indication that they should report data on two clients: a client with a “good” relationship and with a “challenging” relationship, which resulted into each participant filling the instruments twice – one for each case (adapted from Doran et al., 2018). The average time for completing all instruments was 15 minutes. As all questions were mandatory, there were no missing values. This methodology ensured comprehensive data collection and adherence to the study's objectives.

### Data Collection

The sampling method employed was a non-probabilistic snowball technique, utilizing social networks (e.g., Facebook, Linkedin), email, and the researchers' personal contacts for participant recruitment. An invitation post was presented with a link that led to the questionnaire. Some Portuguese Psychotherapy Associations were also contacted by e-mail to disseminate the study through their associates.

To participate, individuals were required to click on the provided link, leading them to the online Google Form containing the informed consent. Upon providing consent, participants proceeded to complete the previously described instruments. All participants met the established criteria for inclusion in the sample of 100 psychologists, and no individuals were excluded from the study.

### Statistical Analyses

First, to explore and to confirm the factorial structure of the ANS-T_Pt, the data from each participant was randomly split into two different sub-samples constituted by 50% of participants data reported to a GTR and the other 50% to CTR. Both random sub-samples have the same therapists; in the first half therapists responded reporting a good relationship and the second half a challenging relationship and vice-versa. The exploratory factor analysis was conducted with the first sub-sample (*N* = 100) and to decide the number of factors a parallel analysis was used. The confirmatory factor analysis was performed in the second random sub-sample (*N* = 100) using maximum likelihood (ML) estimation. Multiple fit indexes were used to analyze model fit (Hooper et al., 2008; Hu & Bentler, 1999): the Chi-square (χ^2^) and the Normed Chi-square (χ^2^/*df*) less than 3, the Comparative Fit Index (CFI > .95), the Tucker–Lewis Index (TLI > .95), the Root Mean Square Error of Approximation and the Standardized Root Mean Square Residual (RMSEA and SRMR ≤ .08). A composite reliability score was assessed to evaluate internal consistency. EFA and CFA were conducted using R software (R Core Team, 2019).

Afterwards, criterion-related validity was investigated through Pearson bivariate correlation analysis to assess the relationship between the ANS-T_Pt and the WAI-T, with therapists’ variables and variables from the therapeutic relationship.

## Results

### Construct Validity

#### Factor Structure and Internal Consistency

**Figure 1:**
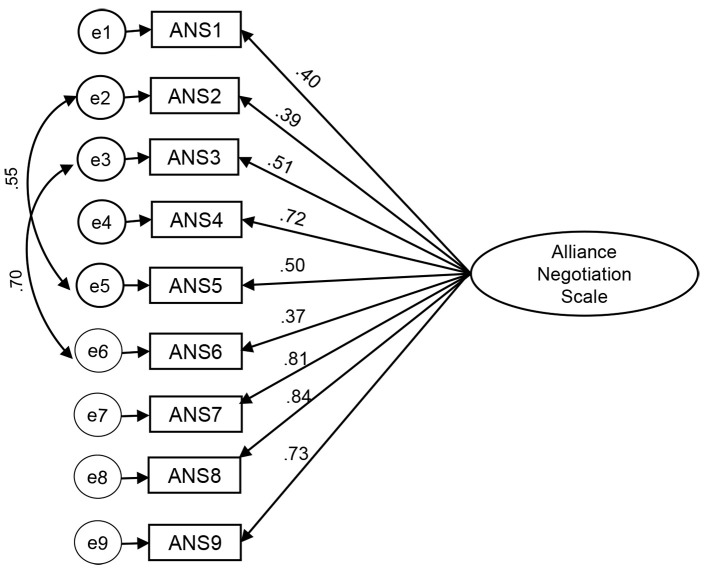
Alliance Negotiation Scale, Confirmatory Factor Model

Firstly, the matrix factorability was supported with a KMO of .80 and the items were significantly correlated, χ^2^(36) = 507.00, *p* < .001. A principal components analysis was conducted and based on parallel analysis, a one-factor solution was obtained, as the original measure (Doran et al., 2018). That factor explained approximately 53% of the total variance. The loadings ranged between .63 and .83. Afterwards, a confirmatory factor analysis was performed in the second random sub-sample (*N* = 100). To the exception of the RMSEA (.09, 95% CI [.05, .14]), all the others goodness of fit indices showed that unifactorial structure had a good fit to the data: χ^2^(24, *N* = 100) = 46.00, *p* = .004, χ^2^/*df* = 1.92; CFI = .94; TLI = .92; SRMR = .07. The standardized factor loadings ranged from .39 to .86 (Figure 1) and all were significant (*p* < .001).

The internal consistency reliability of the ANS-T_Pt was assessed through the composite reliability, and the obtained result demonstrated an adequate reliability (CR = .83, Hair et al., 2019).

### Criterion-Related Validity

A bivariate Pearson correlation between the Portuguese version of WAI-T and ANS-T_Pt showed that the ANS-T_Pt was positively correlated with all the WAI-T, for the sub-scales and the sample (Table 1). However, the relationship between the ANS-T_Pt and the WAI-T were stronger in the challenging relationship (Table 2).

**Table 1:** Correlations Between ANS and WAI for Good Therapeutic Relationship

Variable	1	2	3	4
1. ANS	−	−	−	−
2. WAI Goals	.47*	−	−	−
3. WAI Tasks	.39*	.72*	−	−
4. WAI Bond	.48*	.73*	.66*	−
WAI Total	.50*	.91*	.87*	.90*

*Note*. *N* = 100. ANS = Alliance Negotiation Scale; WAI = Working Alliance Inventory.

*p < .001.

**Table 2:** Correlations Between ANS and WAI for Challenging Therapeutic Relationship

Variable	1	2	3	4
1. ANS	−	−	−	−
2. WAI Goals	.56*	−	−	−
3. WAI Tasks	.56*	.79*	−	−
4. WAI Bond	.47*	.61*	.58*	−
WAI Total	.60*	.91*	.89*	.84*

*Note*. *N* = 100. ANS = Alliance Negotiation Scale; WAI – Working Alliance Inventory.

*p < .001.

Furthermore, through bivariate Pearson correlation we have explored the relationship between the alliance negotiation and therapist features, and the analysis indicate that alliance negotiation in the challenging relationship was positively associated with the therapist years of experience (*r* = .24, *p* = .019) and the mean number of patients per week (*r* = .22, *p* = .026). And the alliance negotiation in the good relationship was associated with the mean number of patients per week (*r* = .24, *p* = .019).

For the challenging relationship, higher alliance negotiation was positively associated to how close the therapist felt to his/her client (*r* = .47, *p* < .001) and how please the therapist felt with the therapeutic work done so far (*r* = .43, *p* < .001).

As for the good relationship cases, higher alliance negotiation was also positively associated to how close the therapist felt to his/her client (*r* = .21, *p* = .039) and how please the therapist felt with the therapeutic work done so far (*r* = .26, *p* = .008). However, these correlations were lower than the other ones.

## Discussion

In this study, we aimed to translate, perform the cultural adaptation and a preliminary psychometric analysis of the Portuguese form of the therapist version of the Alliance Negotiation Scale (ANS-T_Pt). This contribution enhances our understanding of alliance negotiation within Portuguese-speaking psychotherapeutic processes. Moreover, this study provides preliminary evidence for the unifactorial structure of the ANS-T_Pt, its internal consistency, and its criterion-related validity with the working alliance construct.

In terms of construct validity, the Portuguese version exhibited a structure akin to the original version, featuring a single factor with nine items. The Cronbach's alpha coefficient for the Portuguese form of the ANS-T was adequate for both case types and comparable to values reported in prior version (α = .84 English version; α = .82 Spanish version; Doran et al., 2018; Gómez-Penedo et al., 2019).

Furthermore, evidence was found to establish the criterion-related validity of the instrument, based on the correlations between the ANS-T_Pt and WAI-T scores. The results showed moderate to large associations between the instruments (Cohen, 2016), suggesting that both measures are correlated despite measuring different constructs (as previous showed in the English and Spanish versions). Other similar aspect is that the correlations between the ANS-T_Pt and the WAI-T (*r* = .50 and *r* = .60 respectively, *p* < .001), were lower than those observed in the client’s versions (*r* = .72, *p* < .001). As discussed in the Spanish version (Gómez-Penedo et al., 2019), this suggests that in the therapist's version there may be a higher degree of differentiation between the alliance studied as collaboration and the alliance as negotiation. The distinct structure, both in terms of the number of factors and items, may also contribute to these findings.

The measure, in contrast to the client version, comprises a singular factor. The authors (Doran et al., 2018; Gómez-Penedo et al., 2019) suggested that this difference may stem from the perspective shift—while the client's viewpoint considers two facets (degree of client comfort to present negative feelings and flexibility of the therapist), the therapist's perspective presents alliance negotiation as a more encompassing phenomenon. While this proposition holds merit, we advocate for further research to validate this assumption.

Other interesting aspect is that, in our study we tried to overcome previous referred limitations trying to gather information about the client. On the group of cases perceived by the therapist has having a challenging relationship, there was a higher degree of association between WAI-T total scores and its subscales and ANS-T_Pt. Alliance negotiation was associated with the working alliance specially when in presence of a challenging relationship. Noteworthy, is the higher correlation between tasks and goals in the challenging relationships when compared with the good relationships, where this association is lower.

In our study, we have also explored the relationship between the alliance negotiation and therapist’s features. The analysis indicates that the alliance negotiation in the challenging relationship was positively associated with the therapist years of experience and the mean number of clients per week. Considering the nature of the negotiation process, in dealing with alliance ruptures (in more challenging relationships) this result might be explained by the fact that a more experienced therapist may be more capable to deal with these challenges. Also, for the challenging relationship, higher alliance negotiation was positively associated to how close the therapist felt to his/her client and how pleased the therapist felt with the therapeutic work done so far. This result may also be linked to the association observed between the bond and tasks subscales of the WAI-T, where there was a higher association in the challenging relationship cases. Also noteworthy, is the mean number of sessions of this group, 25, which means that even though it was perceived as a challenging relationship it was an enduring one.

As for the cases perceived as having a good relationship, higher alliance negotiation was also positively associated to how close the therapist felt to his/her client and how pleased the therapist felt with the therapeutic work done so far. However, these correlations were lower when compared to the challenging relationship cases, meaning that this may be less associated with the negotiation aspect of the alliance. This may also mean that with challenging relationships therapists may invest more, which may contribute to the closeness of the client and feeling more satisfied with the work. It is not possible to determine whether it is the working alliance that allows for the negotiation or the negotiation that allows for the working alliance. Nevertheless, this result is important because it may capture the nature of the alliance negotiation as a different aspect from the working alliance, even though these are related constructs. The development and negotiation of an alliance is both a critical and pivotal point in the therapeutic process. A key to a successful therapeutic alliance may be the ability of the intervenient to develop a relationship supported by mutual trust and commitment.

### Limitations and Future Research

Despite the usefulness of the present scale, these results may need further investigation. In data collection we asked for good and challenging relationships, not specifically for bad relationships which could lead to different results. A challenging relationship may indeed allow for a more negotiated process but still be a good (enough) one, which can be different from a bad relationship where this negotiation may not even be possible and could even lead to earlier dropouts. Also, and related, is that this was a cross sectional study and data was collected online, with an heterogenous sample regarding the timing of the therapeutic process, and with different number of sessions (ranging from 1 to 160), meaning that the therapeutic alliance was at different stages. With some clients the therapeutic alliance was only beginning, while with others it was a long one. It is possible that the results might have been different with other conditions, such as limited to a particular point in time of the therapeutic process, with a high variability between participants or a representative sample, and data collected in person or immediately following therapy sessions or even considering different case characteristics such as, for example, drop out cases.

In addition, the cross-sectional nature of our study limits the exploration of the stability of the construct and its evolution over time. A longitudinal study utilizing a repeated measures design would enable the examination of fluctuations in therapist perceptions and the evolution of negotiation (ANS) and quality (WAI) of the therapeutic alliance. This approach would provide clarity on whether it is the quality of the working alliance that facilitates negotiation or the degree of negotiation that fosters the quality of the working alliance. The negotiation as a concept appears to re-conceptualize the therapeutic alliance as a continuous negotiation of the needs of two independent subjects involved in the relationship and reflect on how far disagreements and tension are processed by and within the therapeutic process. Therefore, in future studies the self-report measure could be revised to capture this process or be better though to be used in a continuum assessment. Recent studies indicate that the alliance is codeveloped with clients, which reinforces this perspective of the alliance being developed and negotiated rather than a static construct (Escudero et al., 2022). We suggest that more studies are needed regarding its structure and replication with different samples. Given that the client version has two factors, a revision of the measure may be considered to create better symmetry between measures. We would argue that a good measure for assessing the quality of the working alliance would integrate items that capture several aspects such as: quality of the bond, ability to express disagreements, agreement of goals and tasks, negotiation of goals and tasks.

In future studies, it could also be of interest to further study the impact of the therapist characteristics such as age, gender or therapeutic model, and its matching with the client and its impact on the alliance negotiation. While our sample predominantly comprised females (85%), aligning with the gender distribution of psychologists in Portugal (84.2% according to the 2014 census of the Border of Portuguese Psychologists), this gender composition may pose some limitations that warrant further investigation.

### Implications and Contributions

To the best of our knowledge, the ANS is the first measure to assess the negotiation concept using a brief self‐report format. Existing research on the presence of ruptures and their repair traditionally rely on observer‐based coding methods rather than client and therapist self‐report (e.g., Eubanks-Carter et al., 2015). Reinforcing Doran and collaborators (Doran et al., 2018) arguments, although interesting and informative, such methods are costly and time consuming in nature. Being brief and easy to use, may not only contribute to the study of alliance negotiation, but may also be a significant measure for clinical practice and supervision, allowing to use the response to the items has a reflection on the negotiation work with the client.

Our results seem promising, in line with the previous studied versions of the scale and will allow to increase the alliance negotiation studies in Portuguese speaking countries. Meanwhile, to have both versions, for clients and therapist, of ANS will also allow the dyadic study of negotiation. Even if we have come a long way on research regarding the relationship between process and outcome, there remains unexplained variance and critical gaps in our understanding about what processes produce therapeutic change (e.g., Doran et al., 2018; Zilcha-Mano & Ben David-Sela, 2022). It seems useful and necessary to understand the relationship and the impact alliance negotiation has more fully on treatment and treatment outcome.

### Conclusion

This was a preliminary validation of the ANS Therapist Version to Portuguese, showing that this instrument is reliable, valid and a parsimonious measure of the alliance negotiation which allows for the evaluation of the efficacy of the therapeutic processes that can be used in clinical settings and to research purposes.

## Supplementary Materials

The Supplementary Materials contain the following item (for access see Nunes da Silva et al., 2024):

- Escala de Negociação da Aliança Terapêutica (Versão do terapeuta) [Portuguese form of the Alliance Negotiation Scale – Therapist Version]

Nunes da SilvaA. C.
MatosM.
CarvalhoH.
 (2024). Supplementary materials to "The Alliance Negotiation Scale – Therapist Version: Psychometric properties in a sample of Portuguese psychologists"
[Portuguese form of the Alliance Negotiation Scale – Therapist Version (ANS-T_Pt)]. PsychOpen. 10.23668/psycharchives.14181
PMC1130391939119052

## Data Availability

The data that support the findings of this study are available from the corresponding author upon reasonable request.
